# Prevalence and risk factors of colon polyps and other colonic lesions in acromegaly: Insights from colonoscopy screening

**DOI:** 10.1007/s11102-025-01513-4

**Published:** 2025-04-01

**Authors:** Sema Hepşen, Enes Üçgül, Burak Menekşe, Burçak Cavnar Helvacı, Ceren Karaçalık Ünver, Halil Durantaş, Oğulcan Boz, Yusuf Coşkun, Başak Çakal, Muhammed Kızılgül, Erman Çakal

**Affiliations:** 1 Department of Endocrinology and Metabolism, Ankara Etlik City Hospital, Ankara, Türkiye; 2 Department of Gastroenterology, Ankara Etlik City Hospital, Ankara, Türkiye; 3 Department of Gastroenterology, University of Health Sciences, Ankara Training and Research Hospital, Ankara, Türkiye; 4 Endocrine and Diabetes Division, University of Minnesota Twin Cities, Minneapolis, USA

**Keywords:** Acromegaly, Colonoscopy, Colon polyps, Colon lesions, Colon cancer

## Abstract

**Purpose:**

The existing data on colon lesions in acromegaly is notably heterogeneous. This study aimed to analyze the endoscopic and histopathological characteristics of colon polyps and other colonic lesions in acromegaly patients.

**Methods:**

This case-control study included 192 acromegaly patients and 256 controls. Colon polyps were categorized based on their size and histopathological classification. Colon malignancies and other colonic lesions, such as anal fissures, hemorrhoids, and diverticulosis, were also documented.

**Results:**

The prevalence of colon polyps was higher in the acromegaly group than in controls (*p* = 0.003), however, no differences were observed in the number, size, or histopathological subtypes of the polyps. Polyps in acromegaly patients were predominantly located in the distal colon and rectum. Multiple polyp locations and histopathological subtypes were more frequent in the control group (*p* = 0.042 and *p* = 0.018). Rates of low-grade dysplasia, high-grade dysplasia, and malignancy were similar between groups. Anal fissures were more common in the acromegaly group, whereas diverticulosis was less frequent (*p* = 0.001 and *p* < 0.001; respectively). Logistic regression analysis identified no significant clinical or laboratory predictors for colon polyps in acromegaly.

**Conclusion:**

Patients with acromegaly exhibited a higher prevalence of colon polyps, predominantly located in the distal colon, which typically displayed a single histopathological subtype. No increased rates of colonic dysplasia, colon cancer, or other colonic lesions were observed in patients with acromegaly, except for an elevated prevalence of anal fissures.

## Introduction

Acromegaly is an endocrine disorder characterized by excessive growth hormone (GH) and insulin-like growth factor-1 (IGF-1) levels, commonly caused by a pituitary adenoma. This complex condition is associated with various comorbidities, including diabetes mellitus (DM), hypertension, cardiovascular diseases, obstructive sleep apnea syndrome (OSAS), and respiratory problems [1]. The association between acromegaly and increased rates of solid organ malignancies, including colorectal, thyroid, breast, and prostate cancers, remains a subject of ongoing debate, with the literature on this topic being highly heterogeneous [2, 3]. Among the morbidities associated with acromegaly, premalignant and malignant colon lesions remain prominent areas of interest and continue to garner significant attention from researchers due to the lack of definitive consensus in the existing literature.

Elevated IGF-1 levels have been found to promote increased cellular proliferation and anti-apoptotic activity in the colorectal epithelium, which are responsible for the development of colon polyps and malignancies [2]. Despite inconsistencies regarding the increased rate of colon cancer, it is widely accepted that colon polyps are more prevalent in patients with acromegaly compared to the general population [4, 5]. Furthermore, malignancies have become a comparable cause of death to cardiovascular diseases in acromegaly [6, 7]. Therefore, in acromegaly patients, although some recommendations may vary based on age, most current guidelines recommend performing a screening colonoscopy at the time of diagnosis, followed by regular surveillance colonoscopies [8–10]. However, some recent reports have concluded that the incidence of malignancy in acromegaly is decreasing and is now comparable to that of the reference population, likely due to the increased rate of early disease detection and advances in therapeutic options [11]. In line with these findings, some guidelines do not recommend a routine cancer screening program specific to acromegaly [12].

Endoscopic findings, including the size and location of colon polyps in patients with acromegaly, as well as the histopathological classification of polyps, are reported to be heterogeneous in the literature [4, 13]. Additionally, data on the risk factors associated with polyps, such as age, disease duration, obesity, GH and IGF-1 levels, fasting glucose, and insulin levels, show significant variability across studies, and these factors have not yet been sufficiently clarified.

This large-scale, single-center study aimed to conduct a comparative analysis of the endoscopic and histopathological characteristics of colon polyps and other colonic lesions in patients with acromegaly and to identify the risk factors associated with colon polyps.

## Materials and methods

### Study design

This retrospectively designed case-control study was approved by the Ethics Committee of Ministry of Health Ankara Etlik City Hospital in accordance with the principles of the Helsinki Declaration. The written informed consent forms were obtained from all participants.

### Patient inclusion

**Fig. 1:**
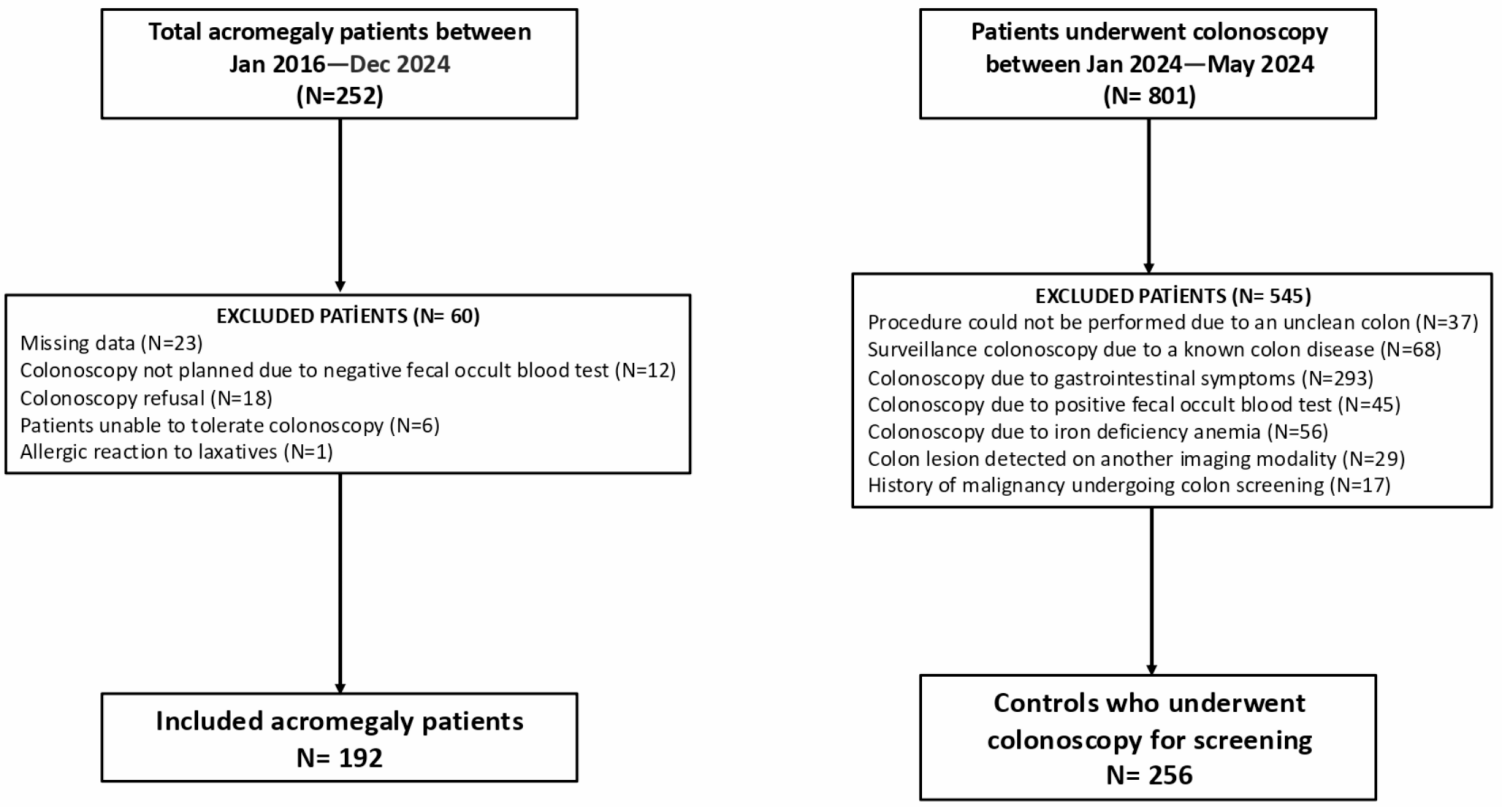
Flow chart of patient inclusion

A total of 252 acromegaly patients followed in our clinic between January 2016 and December 2024 were assessed. Among them, 192 patients who underwent a colonoscopy either at the time of diagnosis or during follow-up were included in the study. The details of the patients who did not undergo a colonoscopy are presented in Fig. 1.

For the control group, consecutive 801 patients who underwent colonoscopy at the outpatient gastroenterology unit of our hospital between January 2024 and May 2024 were evaluated. Patients who underwent colonoscopy due to gastrointestinal complaints, a positive fecal occult blood test, detection of a colon lesion on another imaging modality, a diagnosis of iron deficiency, a known malignancy, or surveillance for a known colon disease were excluded from the study. Additionally, patients in whom the procedure could not be completed due to inadequate bowel preparation were also excluded. The remaining 256 patients who underwent colonoscopy for screening purposes constituted the control group. The flowchart of patient inclusion is presented in Fig. 1.

### Baseline data

Demographic data and comorbidities, such as DM, hypertension, coronary artery disease, and OSAS, were recorded for the participants. IGF-1 and GH levels, as well as the size of the pituitary adenoma at the time of diagnosis were also documented. The initial treatment of the patients, whether surgery or somatostatin analogue, was recorded.

### Colonoscopy evaluation

Bowel preparation adequacy data was recorded for all participants. Colonic lesions were classified into four categories: colon polyps, colon dysplasia, malignant lesions, and perianal diseases, which included anal fissures, hemorrhoids, and diverticulosis. Colon polyps were further classified based on their histopathological results using the World Health Organization classification as neoplastic and non-neoplastic polyps [14]. Neoplastic polyps included tubular, villous, and tubulovillous adenomas, while non-neoplastic polyps included inflammatory, hamartomatous, and hyperplastic polyps [15, 16]. Dysplasia was classified into low-grade and high-grade dysplasia [14]. Polyp size is categorized based on the size of the largest polyp as < 5 mm, 5–10 mm, and ≥ 10 mm, in accordance with the 2024 European Society of Gastroenterology Guideline [17].

### Statistical analysis

The normality of variables was evaluated using the Kolmogorov-Smirnov and Shapiro-Wilk tests. Comparisons of categorical variables were performed using the Chi-square test, or Fisher’s exact test when the Chi-square assumptions were not met due to low expected frequencies. The Mann-Whitney U test was used for comparisons of nonparametric variables between acromegaly patients and controls. Categorical variables were presented as numbers and percentages, while non-normally distributed variables were reported as medians with interquartile ranges (IQR, 25th–75th percentile). Spearman’s correlation coefficients and their significance were used to analyze the associations between the number of colon polyps and other variables in both groups. Univariate analysis was performed to identify potential factors for inclusion in multivariable logistic regression analysis to determine the final predictive factors for colon polyp presence in acromegaly patients. Model fit was assessed using the Hosmer-Lemeshow goodness-of-fit test. A 5% type I error level was used to infer statistical significance, and p-values less than 0.05 were considered statistically significant for all tests.

## Results

### Baseline data

A total of 192 patients with acromegaly and 256 controls were analyzed. Age and sex distributions were comparable between the groups (*p* = 0.09 and *p* = 0.231, respectively). Baseline characteristics, comorbidities, and acromegaly-specific data are summarized in Table 1.

**Table 1:** Baseline data belonging to acromegaly patients and controls

	Acromegaly (*N* = 192)	Controls (*N* = 256)	*P* value
Age, years	54 (42–63)	55 (48–64)	0.09
Female sex, n (%)	105 (54.6)	134 (52.34)	0.231
BMI, kg/m^2^	29.4 (26.6–33.2)		
Smoking, n (%)	49 (25.5)	44 (17.1)	0.069
**Comorbidities**			
Diabetes mellitus, n (%)	55 (28.6)	57 (22.3)	0.116
Hypertension, n (%)	78 (40.6)	56 (21.8)	**< 0.001**
Coronary artery disease, n (%)	19 (10)	26 (10.1)	0.244
OSAS, n (%)	26 (13.5)	4 (1.6)	**0.002**
**Data belonging to acromegaly**			
Initial adenoma size, mm	15 (10–25)		
IGF-1 level at the diagnosis time, ng/mL	657 (512–901)		
GH level at the diagnosis time, mcg/L	9.2 (4.9–21.6)		
Somatostatin analogue for initial treatment, n (%)	15 (7.9)		
Surgery for initial treatment, n (%)	177 (92.2)		

BMI: Body mass index, IGF-1: Insulin like growth factor-1, GH: Growth hormone, OSAS: Obstructive sleep apnea syndrome

Categorical data are presented as numbers and percentages, non-parametric data are presented as medians (IQR 25–75)

**Table 2:** Data belonging to detected colon polyps and other colon lesions in patients with acromegaly and controls

	Acromegaly (*N* = 192)	Controls (*N* = 256)	*P* value
Patients with detected polyp, n (%)	72 (37.5)	64 (25)	**0.003**
Polyp number, median (IQR)	2.25 (1–2)	2 (1–2)	0.273
Polyp size of the largest polyp*			0.233
< 5 mm, n (%)	56 (77.8)	43 (67.2)	
5–10 mm, n (%)	11 (15.3)	11 (17.2)	
≥ 10 mm, n (%)	5 (6.9)	10 (15.6)	
**Patients with neoplastic polyps, n (%)**	37 (19.2)	41 (16)	0.653
Tubular adenoma, n (%)	33 (17.1)	41 (16)	0.039
Villous adenoma, n (%)	2 (1)	6 (2.3)	0.998
Tubulovillous adenoma, n (%)	6 (3.1)	7 (2.7)	0.772
**Patients with dysplasia, n (%)**			
Low grade dysplasia, n (%)	1 (0.5)	3 (1.2)	1.00
High grade dysplasia, n (%)	1 (0.5)	4 (1.6)	0.369
**Patients with non-neoplastic polyps, n (%)**			
Inflammatory polyp, n (%)	2 (1)	6 (2.3)	1.00
Hyperplastic polyp, n (%)	36 (18.8)	24 (9.4)	0.120
**Malign sitology, n (%)**	1 (0.5)	1 (0.4)	1.00
**Other lesions, n (%)**			
Anal fissure, n (%)	4 (2.1)	1 (0.4)	**0.001**
Hemorrhoids, n (%)	38 (20)	60 (23.4)	0.419
Diverticulosis, n (%)	5 (2.6)	22 (8.6)	**< 0.001**

*The percentages for polyp size have been provided among the detected polyps

### Comparative analysis

Among the acromegaly patients, colonoscopy was performed at the time of diagnosis in 179 patients (93.2%) and during follow-up in 13 patients (6.8%). Adequate bowel preparation was achieved in 141 patients (73.4%) in the acromegaly group and 210 patients (82%) in the control group (*p* = 0.028).

**Fig. 2:**
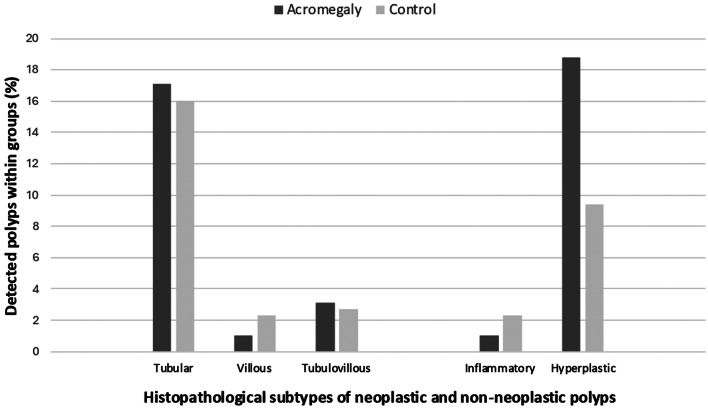
Histopathological subtypes of detected colon polyps

**Fig. 3:**
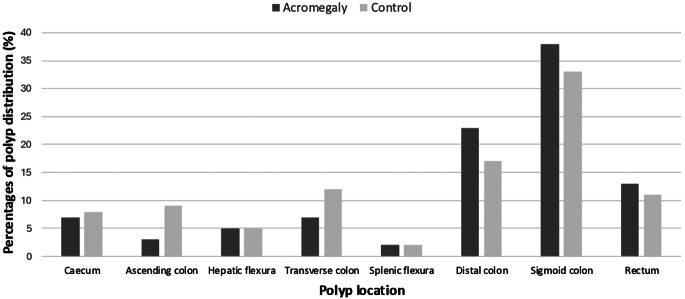
The distribution of colon polyp localization through colon segments

The prevalence of colon polyps was higher in the acromegaly group compared to the control group, with 72 patients (37.5%) in the acromegaly group and 64 patients (25%) in the control group (*p* = 0.003). The number and size of polyps were similar between the two groups (*p* = 0.273 and *p* = 0.233, respectively). The prevalence of colon polyps was higher in the acromegaly group among patients with adequate colon cleansing (*p* = 0.007). However, when partial colon cleansing was achieved, polyp rates were similar between the acromegaly and control groups (*p* = 0.679). No differences were observed in the subtypes of neoplastic and non-neoplastic polyps between the groups (*p* > 0.005 for each). The details of the endoscopic and histopathological characteristics are presented in Table 2. Figure 2 shows the percentages of colon polyp subtypes across the entire groups. Colon polyps in patients with acromegaly tended to be located in the distal segments of the colon and rectum. However, no differences were observed in the distribution of polyps across specific colon segments in the comparative analysis (*p* > 0.005 for each). The distribution of colon polyp localization through colon segments is illustrated in Fig. 3.

Among patients with colon polyps, multiple polyp settlements were observed more frequently in the control group compared to the acromegaly group (17 [23.6%] vs. 26 [40.6%], *p* = 0.042). Similarly, multiple histopathological subtypes of colon polyps were more common in the control group than in the acromegaly group (6 [8.3%] vs. 15 [23.4%], *p* = 0.018).

Rates of low-grade dysplasia, high-grade dysplasia, and malignancy were similar between patients with acromegaly and the control group (*p* > 0.005 for each). Anal fissures were more frequently detected in the acromegaly group compared to the control group, whereas diverticulosis was less common (*p* = 0.001 and *p* < 0.001; respectively). The rate of hemorrhoids was similar across the groups (*p* = 0.419).

### Correlation and regression analysis

In the correlation analysis, age was found to be positively correlated with the number of colon polyps in the control group (*r* = 0.189, *p* < 0.001), whereas no correlation was observed with sex. However, no relationship was found between the number of colon polyps and age, sex, body mass index (BMI), adenoma size, basal GH, or IGF-1 levels in the acromegaly group (*p* > 0.005 for each).

Univariate analysis suggested that the presence of DM was associated with the presence of colon polyps in acromegaly patients (*p* = 0.037, R² = 0.025). However, sex, BMI, the presence of OSAS, smoking, basal IGF-1, and GH levels were not found to be associated.

Based on these findings, a multivariable logistic regression model was conducted to analyze the predictive factors for the presence of colon polyps in acromegaly, including age, sex, DM, and basal IGF-1 and GH levels. None of these parameters were identified as independent predictive factors for polyp presence in patients with acromegaly.

## Discussion

This study confirmed that the prevalence of colon polyps is higher in patients with acromegaly compared to controls. However, the number and size of polyps were found to be similar to those observed in the control group. Colon polyps in patients with acromegaly were more commonly located in the distal segments of the colon. Additionally, polyps in acromegaly tended to manifest as a single histopathological subtype located in a specific region of the colon, rather than as multiple localized lesions or varied subtypes. While age was positively associated with the number of colon polyps in the control group, neither age nor any other clinical or laboratory factor was associated with polyp number in acromegaly patients. Other colon lesions, including malignancy and dysplasia rates apart from anal fissures, do not appear to be increased in acromegaly.

Sporadic forms of colon cancer develop on the basis of various epigenetic and genetic changes, leading to cell proliferation and differentiation [18]. This process first results in the formation of neoplastic colon adenomas, which subsequently transform to dysplasia and colon cancer [19]. However, in patients with acromegaly, the development of colon polyposis and its malignant transformation is distinct from non-acromegalic colon tumorigenesis [19]. Excessive GH and IGF-1 secretion are postulated to play pivotal roles in stimulating cell proliferation, promoting angiogenesis, and increasing the risk of mutations [20, 21].

The increased rate of colon polyps in acromegaly has been confirmed by many studies and is widely accepted by experts [22, 23]. However, reported endoscopic characteristics, such as polyp number, size, and localization, show significant alterations. Current epidemiological data show that, in the general population, the proximal colon, distal colon, and rectum each account for approximately one-third of colon polyps, with their prevalence increasing with age [24]. Several studies have shown that the localization of colon polyps in acromegaly differs from that in the general population, with polyps more frequently located on the right side of the colon and tended to be larger than 10 mm [4, 25]. Despite these findings, some recent studies have reported a higher rate of polyps in the descending colon [26, 27]. A study involving 178 patients with acromegaly reported findings were consistent with those of the present study, with polyps predominantly located in the sigmoid colon and rectum [13]. However, in their study, polyp size and number were greater in patients with acromegaly. In contrast, the findings of the current study demonstrated that the size and number of colon polyps in acromegaly patients were similar to those in the control group. Additionally, our findings introduced a novel contribution to the literature, suggesting that colon polyps in acromegaly tended to localize to a specific part of the colon rather than involving multiple segments. These results may be explained by the fact that most colonoscopies in our cohort were performed at the time of diagnosis, which allowed for the detection of polyps while they were smaller in size and fewer in number, during the early stages of the disease.

The histopathological characteristics of colon polyps in acromegaly remain controversial, as many studies have not used the standardized classification systems outlined in pathological guidelines. A meta-analysis involving 701 acromegaly patients concluded that both hyperplastic polyps and adenomas were more common in acromegaly patients [2]. However, a detailed analysis of each histopathological subtype was not provided due to the heterogeneity of the included studies. A single-center study involving Turkish patients reported that hyperplastic polyps were more common in acromegaly patients, while other histopathological subtypes were not [28]. Another study indicated that advanced histopathological findings, such as tubulovillous adenomas, were more commonly observed in acromegaly patients than in controls [4]. No significant differences among the histopathological subtypes were identified in our cohort. However, similar to polyp localization, the histopathological subtype of colon polyps in acromegaly also tends to be a unique subtype rather than encompassing multiple subtypes.

Independent factors such as advanced age, smoking, obesity, insulin resistance, dyslipidemia, and DM have been found to be associated with colon carcinogenesis in non-acromegaly patients [29]. Several studies have explored the effects of these factors on premalignant and malignant colon lesions in acromegaly. However, the literature presents extremely heterogeneous results regarding the factors associated with colon polyps, primarily due to variations among acromegaly patients included in these studies. In an Italian cohort, colon polyps were found to be positively associated with GH, IGF-1, fasting glucose, and insulin levels (8). Peng et al. demonstrated an independent association between the presence of colon polyps and both GH adenoma volume and IGF-1 levels [22]. An observational study including 210 acromegaly patients was found a strong relationship with insulin levels and colon polyps [30]. Another retrospective observational study also found an increased risk of polyps in patients with a history of previous polyps and higher IGF-1 levels [31]. In contrast, Renehan et al. indicated no association between colon neoplasms and GH or IGF-1 levels, as well as disease duration [4]. Gonzales et al. found that the prevalence of colon polyps was higher among acromegaly patients with DM [26]. Considering the findings of these previous studies, IGF-1 levels, the presence of DM, and glucose metabolism parameters, such as insulin levels, emerged as prominent factors among all potential associations [32]. The present study demonstrated a positive association between DM and colon polyps in the acromegaly group; however, this association was not maintained in the multivariable regression analysis. Our finding confirmed that age is not an independent risk factor for colon polyps in acromegaly, unlike in controls. All guidelines, except those of the British Society of Gastroenterology, recommend performing an initial colonoscopy at the time of diagnosis [8–10, 33]. In contrast, the recent Danish guideline opposes routine colonoscopy screening in acromegaly and recommends following the general national screening guidelines [12]. All recent study data, including ours, may highlight the need for updated recommendations in the future guidelines.

Previous studies have identified an increased risk of colon cancer in patients with acromegaly [3, 34]. A recent study involving 70 acromegaly patients concluded that while the rate of low-grade dysplasia was similar, the rate of high-grade dysplasia was higher in their acromegaly cohort [27]. In contrast to these findings, some recent studies and reviews have concluded that the incidence of colon cancer, as well as low- and high-grade dysplasia, in acromegaly is not higher than in the general population (11). The similar rates of colon cancer and dysplasia in acromegaly and control groups observed in the present study align with the findings of these latest reports.

Contrary to these numerous studies focusing on colon polyps in acromegaly, data on other colonic lesions is limited. A few studies reported an increased rate of diverticulosis in acromegaly, which contrasts with our findings [8, 28]. The increased rate of anal fissures in the present acromegaly cohort, compared to controls, is a new finding that contributes to the literature.

Lastly, due to slower colon transit in acromegaly, insufficient colon preparation is more frequently observed in acromegaly patients compared to the general population [23]. Slow transit and longer colon length are the primary reasons why total colonoscopy is preferred over sigmoidoscopy in patients with acromegaly [19]. Our findings were consistent with this observation, as a higher rate of colon polyps was noted in acromegaly patients with adequate colon cleansing, while those with insufficient colon preparation showed rates similar to the control group.

The limitations of this study include its retrospective design and single-center setting. Although strict exclusion criteria were applied, and only patients who underwent colonoscopy for screening purposes were included, a potential selection bias cannot be completely ruled out in the control group. However, considering the sample size, a key strength of the study is its position as one of the largest single-center studies in the field. Additionally, endoscopic characteristics and histopathological results were assessed according to the most recent classifications.

In conclusion, the rate of colon polyps was higher in patients with acromegaly, regardless of age, gender, presence of DM, adenoma size, or IGF-1 and GH levels in the present cohort. The polyps in acromegaly tended to be located in the distal parts of the colon and to present as a unique histopathological subtype rather than exhibiting multiple distinct subtypes. Patients with acromegaly are not considered to have a higher risk of colonic dysplasia, colon cancer, or other colonic lesions, except for anal fissures.

## Data Availability

The datasets used and/or analysed during the current study available from the corresponding author on reasonable request.
